# Editorial: Precision medicine: recent advances, current challenges and future perspectives

**DOI:** 10.3389/fphar.2024.1439276

**Published:** 2024-06-07

**Authors:** Oriana Awwad, Mamoun Ahram, Francesca Coperchini, Mariam Abdel Jalil

**Affiliations:** ^1^ Department of Biopharmaceutics and Clinical Pharmacy, School of Pharmacy, The University of Jordan, Amman, Jordan; ^2^ Department of Physiology and Biochemistry, School of Medicine, The University of Jordan, Amman, Jordan; ^3^ Department of Internal Medicine and Therapeutics, University of Pavia, Pavia, Italy

**Keywords:** precision medicine, personalized medicine, pharmacogenetics, gene variants, pharmacologic targets, genotyping

Unlike most medical treatments obtained from large-scale studies and designed as “one-size-fits-all” approach, precision medicine is a modern concept of personalized treatment that aims to provide patients with tailored medical interventions to each individual characteristics (Beckmann and Lew, 2016; Akhoon, 2021; Marques et al., 2024). Precision medicine is advantageous in being dynamic, evolving, and wholesome as it accounts for patients’ medical history, molecular profiles, lifestyle, and external factors within their environment (Faulkner et al., 2020; Denny and Collins, 2021). This approach is designed to facilitate enhanced screening, earlier disease detection, more precise diagnosis, and targeted treatment, allowing patients to receive therapies that work best for them for a more effective and safer clinical management and a more efficient healthcare system (Beckmann and Lew, 2016; Faulkner et al., 2020; Akhoon, 2021; Wang and Wang, 2023).

This patient-centered clinical approach needs to identify specific disease subtypes and to classify patients into various subpopulations that differ in their response and susceptibility to traditional treatments (Wang and Wang, 2023; Marques et al., 2024). The diversity of pharmacological mechanisms and targets can permit therapies to efficiently treat particular clinical patients.

Despite the potential of precision medicine and the growing number of customized drugs, there is a need for evidence of the clinical value of integrating such treatments into healthcare systems. Efforts are also needed to determine the best medical intervention for each patient and medical condition. This Research Topic aims to tackle these issues.

The review by Tavazzani et al. identifies the major challenges in diagnosing and treating inner ear diseases. These include access to the inner ear and difficulty in sampling inner ear fluids. The authors then discuss the possible roles of innovative technologies to overcome such challenges. Combining microneedles, nanocarriers, and gene therapy holds great potential in revolutionizing and personalizing the treatment of inner ear diseases of different etiologies.

Biswas et al. reviewed drugs used in autism spectrum disorder (ASD), focusing on their pharmacokinetics, pharmacodynamics, and genetic and non-genetic factors affecting efficacy and safety. They found that *CYP2D6* and *DRD2* gene variants were associated with an increased risk of hyperprolactinemia in children taking risperidone. The review also discusses the interactions of ASD drugs with other drugs, such as enzyme inducers or inhibitors, and their effects on drug safety and efficacy. In addition, factors that can hinder the implementation of pharmacogenetics are highlighted.

Biswas et al. also introduce the pharmacogenetics and repurposing of COVID-19 therapies. The authors call to undertake a pharmacogenetic assessment of some drugs, particularly those that target certain CYP/transporters, for a safer, more effective management of COVID-19. The authors also promote the application of computational studies to discover new medications.

Dakilah et al. identify CDC25 phosphatases as promising candidates for therapeutic intervention of cancer due to their biological role in activating cyclin-dependent kinases and, hence, regulating the cell cycle. The review summarizes the evidence related to the dysregulation of CDC25 phosphatases in various types of cancer. In addition, the authors offer an overview of the enzyme genetic variants underlying the importance of CDC25 inhibition as a therapeutic target for individualized cancer treatment.

Shubbar et al. present *CYP2C19*, a gene involved in the metabolism of commonly prescribed medications, as an interesting target for optimizing healthcare provision through precision medicine. The authors demonstrate how genetic variations of *CYP2C19* can play a role in the metabolism of certain drugs affecting the disease-related therapeutic outcomes. The review then addresses *CYP2C19* genotyping as an opportunity to promote drug efficacy and safety with clinical significance in various medical conditions.

Birla et al. describe the principle of polypills, explore their evidence-based strengths and weaknesses in the management of cardiovascular medicine, elaborate on their potential use in the prevention and treatment of cardiovascular diseases, and list relevant clinical trials involving polypills. The review found that polypills can reduce major adverse cardiovascular events, improve medication adherence, and lower healthcare costs. However, challenges include dosage adjustment, acceptability, and the need for more safety studies. Further research is required to assess customizing polypills for personalized therapeutics.

Greco et al. report drug repositioning and its importance in treating thyroid cancer. The authors offer an overview of the anti-diabetic drugs and their anticancer activities. An example is metformin. In drug repositioning, understanding the disease-drug relationship is crucial. This involves using various approaches to discover disease-gene, disease-disease, and disease-target relationships.

The paper by Cavalloro et al. identifies novel multi-target ligands for managing neurodegenerative diseases using a combination of computational and experimental methods. The ligands influence the HuD/brain-derived neurotrophic factor (BDNF) pathway and activate the ubiquitin-proteasome system via two synergistically acting mechanisms. The outcome of this study promotes precision medicine as a powerful tool for managing neurodegenerative diseases. Yet, future research is needed to overcome the related challenges.

As an attempt to integrate genomics into community health, David et al. report the evolution, strategy, and implementation of the NorthShore University HealthSystem, which is an integrated, personalized healthcare delivery system that involves several hospitals and multispecialty group practice of over 140 locations. The system required the development of screening tools, integrated pharmacogenomics programming, educational programming, electronic medical record integration, and robust clinical decision supportive tools. Over 100 primary care providers were trained in genomic medicine, and more than 225,000 patients were screened for hereditary conditions. Over 4,000 patients have been identified to have genetic variations with medical management implications. The successful experience of NorthShore can be applied to other communities.

These studies highlight the importance of collective knowledge of the diverse pharmacological strategies, mechanisms, and targets as well as the in-and-out details of patients for the success of treatments. Such integration may also ultimately lead to changes in the regulatory, ethical, and policy domains of drug therapy (Faulkner et al., 2020; Marques et al., 2024).

## References

[B1] Akhoon N. (2021). Precision medicine: a new paradigm in therapeutics. Int. J. Prev. Med. 12, 12. 10.4103/ijpvm.IJPVM_375_19 34084309 PMC8106271

[B2] Beckmann J. S. Lew D. (2016). Reconciling evidence-based medicine and precision medicine in the era of big data: challenges and opportunities. Genome Med. 8, 134. 10.1186/s13073-016-0388-7 27993174 PMC5165712

[B3] Denny J. C. Collins F. S. (2021). Precision medicine in 2030-seven ways to transform healthcare. Cell 184 (6), 1415–1419. 10.1016/j.cell.2021.01.015 33740447 PMC9616629

[B4] Faulkner E. Holtorf A. P. Walton S. Liu C. Y. Lin H. Biltaj E. (2020). Being precise about precision medicine: what should value frameworks incorporate to address precision medicine? A report of the personalized precision medicine special interest group. Value Health 23 (5), 529–539. 10.1016/j.jval.2019.11.010 32389217

[B5] Marques L. Costa B. Pereira M. Silva A. Santos J. Saldanha L. (2024). Advancing precision medicine: a review of innovative *in silico* approaches for drug development, clinical Pharmacology and personalized healthcare. Pharmaceutics 16, 332. 10.3390/pharmaceutics16030332 38543226 PMC10975777

[B6] Wang R. C. Wang Z. (2023). Precision medicine: disease subtyping and tailored treatment. Cancers (Basel) 15 (15), 3837. 10.3390/cancers15153837 37568653 PMC10417651

